# Palliative Surgery and Potential Curative Trajectory in a Nasopharyngeal Cancer Survivor With Isolated Lung Metastasis

**DOI:** 10.7759/cureus.69196

**Published:** 2024-09-11

**Authors:** Achyuth Panuganti, Pallvi Kaul, Rahul Kumar, Pankaj K Garg

**Affiliations:** 1 ENT and Head-Neck Surgery, MediCiti Institute of Medical Sciences, Secunderabad, IND; 2 Surgical Oncology, Shri Guru Ram Rai Institute of Medical and Health Sciences, Dehradun, IND; 3 Surgical Oncology, All India Institute of Medical Sciences, Rishikesh, Rishikesh, IND

**Keywords:** lung metastasis, metastatectomy, nasopharyngeal cancer, prognosis, survival

## Abstract

The role of palliative surgery for excision of metastasis in a patient with nasopharyngeal carcinoma (NPC) is limited. However, judicious patient selection can yield noteworthy long-term survival outcomes. We report a case of the occurrence of isolated lung metastasis and its management in a NPC survivor with a long disease-free interval. The patient underwent radiotherapy and chemotherapy in 2014 for primary NPC cT2N1M0. In November 2019, he presented with a cough and respiratory distress. The investigation unveiled an isolated lung metastasis that partially encased the left upper lobe bronchus and closely abutted the left main bronchus and left pulmonary artery. Following comprehensive multidisciplinary consultations involving the patient and relatives, the patient underwent a left pneumonectomy as an imperative palliative intervention to alleviate the symptomatic respiratory distress and long-term disease control. Remarkably, the patient's disease-free status has persisted post surgery until 2024, evoking consideration of a potential curative trajectory. This case emphasises comprehensive evaluations, multidisciplinary discussions, and individualised treatment plans. It encourages patients to remain optimistic and engaged in their healthcare journey.

## Introduction

Nasopharyngeal carcinoma (NPC) accounts for 0.7% (129,079) of new cancer cases and is responsible for 72,987 (0.8%) of cancer-related deaths, as per Globocan 2018 [1-3]. NPC has an unbalanced geographical distribution, with Southeast Asian countries accounting for 67% of the global burden of cancer. Apart from geographical variation, the predominance of disease in certain ethnic groups and male gender predilection are other features of this cancer [4].

Unlike other squamous cell carcinomas of the head and neck region, NPC has a higher tendency for systemic dissemination [5-7]. Nearly 4.4-6% of the patients are detected to have systemic metastases at initial presentation, with skeletal metastases being most common followed by pulmonary and hepatic. A treatment failure rate of 15-42% has been reported in the literature due to distant metastases [8]. Chemotherapy offered with palliative intent remains the mainstay of treatment. However, level 4 evidence exists for surgery and radiofrequency ablation as an alternative modality for the treatment of metastatic NPC.

## Case presentation

In November 2019, we received a referral for a 44-year-old gentleman who had previously survived nasopharyngeal carcinoma. The patient's main concerns were a persistent cough, progressively worsening respiratory distress, and significant weight loss over three months. He stated that he had never consumed alcohol or tobacco in his life.

The patient had been diagnosed with cT2N1M0 SCC nasopharynx in another hospital in December 2013. He had undergone concurrent chemoradiotherapy followed by adjuvant chemotherapy, which was completed in July 2014. Unfortunately, the exact details of chemoradiotherapy could not be retrieved. Subsequently, the patient remained disease-free and was under regular surveillance.

**Figure 1:**
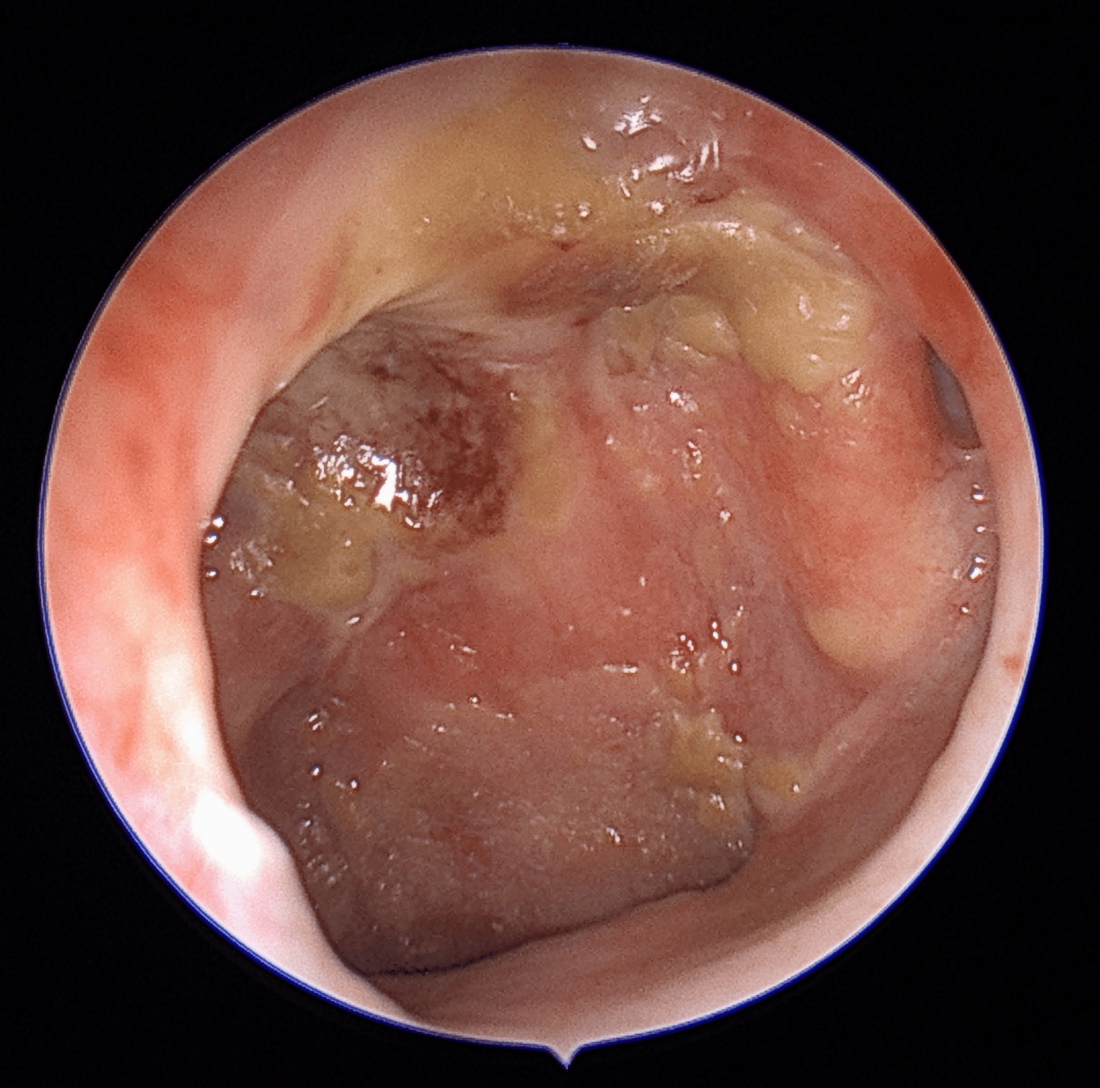
Nasopharyngoscopy shows post-radiation changes with no evidence of local disease recurrence

**Figure 2:**
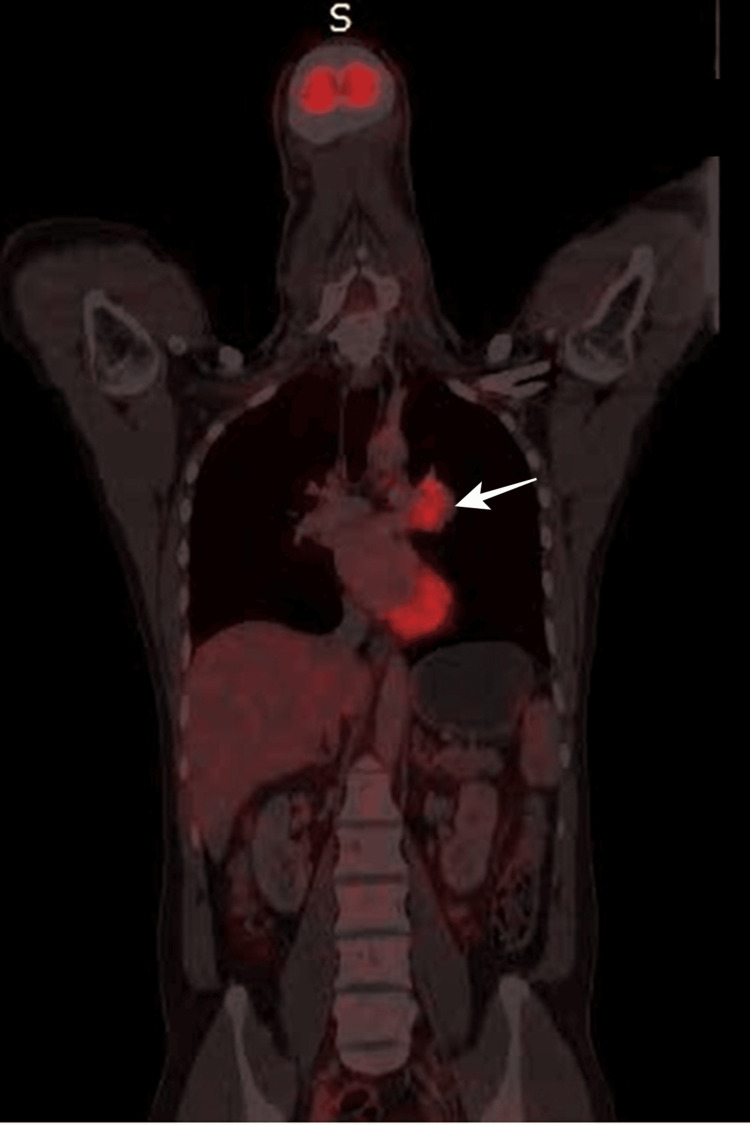
Whole-body PET-CT shows an Isolated FDG avid mass (white arrow) in the left hilum of lung FDG: fluorodeoxyglucose

**Figure 3:**
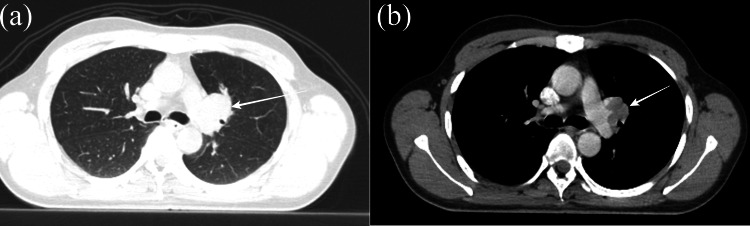
(a) Axial lung window (b) axial mediastinal window of CT scan displays a perihilar mass (white arrow) abutting the left main bronchus and left pulmonary artery

During the evaluation at our hospital, the patient reported being on antihypertensive medication for eight years and receiving thyroid replacement therapy (25 mcg/day of Eltroxin) for four years. He exhibited a good performance status, indicated by an Eastern Cooperative Oncology Group (ECOG) performance status of 1. There were no palpable lymph nodes detected during the neck examination. Nasopharyngoscopy revealed post-radiation changes without any signs of local recurrence (Figure 1). Further imaging investigations, including a whole-body PET-CT scan (Figure 2) and contrast-enhanced CT (Figure 3) revealed a fluorodeoxyglucose (FDG)-avid soft tissue density lesion in the hilar region of the left lung, causing partial encasement of the left upper lobe bronchus and closely abutting the left main bronchus and left pulmonary artery. No other abnormal FDG activity was observed in the nasopharynx or elsewhere in the body.

Bronchoscopic examination confirmed the presence of an endobronchial growth in the left upper lobe bronchus, and a biopsy of the lesion showed large atypical cells with a syncytial arrangement surrounded by lymphocytes. Immunohistochemistry testing demonstrated strong diffuse cytoplasmic positivity for pan-cytokeratin (pan-CK) and focal weak positivity for Epstein-Barr virus latent membrane protein 1 (EBV-LMP-1), consistent with metastasis originating from nasopharyngeal carcinoma.

The case was discussed in the institutional multidisciplinary tumour board, and a decision was made to proceed with surgery primarily to alleviate the symptomatic respiratory distress and with possible long-term disease control. The surgery was offered as a treatment option with a curative intent due to the presence of isolated metastasis, the patient's young age, good performance status, and a long disease-free interval. The option of immunotherapy and palliative chemotherapy was discussed; however, it was not pursued due to the limited scientific evidence supporting immunotherapy in metastatic nasopharyngeal carcinoma and financial constraints given the high cost of immunotherapy. Additionally, the treatment would have been intended only for palliative purposes.

**Figure 4:**
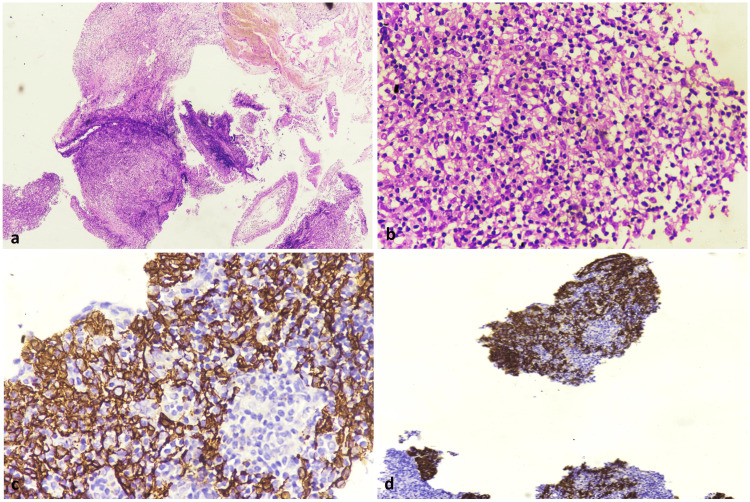
Sections show: (a) Fragments of respiratory mucosa with a tumour in the submucosal region composed of atypical cells in syncytium (H&E 40x); (b) Numerous reactive lymphoid cells in the background (H&E 400x); (c) Atypical cells are immunopositivity for pan cytokeratin (400x); (d) Immuonopositivity for Epstein-Barr virus latent membrane protein 1 (100x)

The patient and caregivers were extensively counselled about the procedure, potential complications, and expected outcomes in their native language, and written informed consent was obtained. Preoperative pulmonary function tests indicated a good pulmonary reserve, making the patient a suitable candidate for lung resection. During the surgery, a 4x4 cm firm mass was found located in the left hilum, encasing the left pulmonary artery and left main bronchus, and was densely adherent to the pericardium. A left pneumonectomy was undertaken to achieve margin-negative resection; the pericardium was opened, and the intra-pericardial segment of the left pulmonary artery was ligated and divided. The postoperative period was uneventful, and the thoracostomy tube was removed on the second postoperative day. The final histopathology report confirmed metastasis originating from nasopharyngeal carcinoma, with clear margins (Figure 4).

The patient was scheduled to receive adjuvant chemoradiotherapy in February 2020 due to the presence of positive mediastinal nodes. However, the treatment was deferred owing to the ongoing COVID-19 pandemic. As of the latest follow-up in the first week of August 2024, the patient has been doing well and is disease-free.

## Discussion

NPC is a common cancer affecting Southeast Asian populations. The non-keratinizing undifferentiated subtype accounts for more than 97% of cases and is strongly associated with EBV infection [8]. Systemic disease relapse is recognised as a major cause of treatment failure in about 15-42% of patients. Skeletal metastases are the most commonly reported, followed by pulmonary and then hepatic metastases [5,7,9,10]. In our patient, the long disease-free interval between the treatment completion for the primary nasopharyngeal tumour and the appearance of a distant metastatic lung lesion five years later without any radiological or clinical evidence of disease in the nasopharynx raised a possibility of a second primary tumour of the lung. However, histopathological examination and immunohistochemistry (EBV-LMP-1 positivity) of the endobronchial biopsy confirmed NPC metastasis [6]. 

Chemotherapy remains the mainstay of treatment for metastatic NPC but is palliative and does not result in survival benefits. Although no randomised trial has been reported comparing the different chemotherapy regimens, platinum-based regimens are optimal first-line systemic therapy. Monotherapy with drugs such as gemcitabine, capecitabine, or taxanes is a widely used palliative chemotherapy regimen with acceptable results. Targeted therapy has also been proposed as a treatment option in light of the over-expression of Epidermal Growth Factor Receptor 1 (EGFR1) and C-kit [11]. Alternative treatment options that have been suggested include surgery and radiofrequency ablation [12]. 

Pulmonary metastasectomy is a recognised therapeutic modality for the treatment of pulmonary metastasis. However, there is a lack of consensus on its role in head and neck cancers. A retrospective review of 21 patients who underwent resection of pulmonary metastases from head and neck malignancies highlighted five- and ten-year overall survival rates of 67% and 55%, respectively, in a selected group of patients [13]. Another retrospective review of 58 patients with pulmonary metastasis of head and neck cancer undergoing surgery highlighted three-year and five-year survival rates of 54.2% and 35.7%, respectively, with a median survival time of 42.2 months [14]. A systematic review of 403 patients with head and neck squamous cell carcinoma who underwent pulmonary metastasectomy confirmed survival benefits and provided level 2a evidence [15]. However, literature assessing the feasibility of metastasectomy for metachronous pulmonary metastases in NPC is sparse. Lim et al. reported four patients with pulmonary metastases who underwent curative resection and concluded that a metastasectomy is a feasible option for treating metachronous and resectable oligometastatic NPC in the lung [8]. Cheng et al. reported a two-year survival rate of 80% in 12 patients who underwent surgical resection when compared with a historical control group consisting of 65 patients who received chemotherapy with a mere survival rate of 24.1% [16].

Careful selection of patients for metastasectomy is imperative for good oncological outcomes. Various patient selection criteria for determining the indication of surgery have been proposed, and the one followed at our institution is Thomford’s criteria. It assesses the following parameters/ prerequisites: (a) general status capable of undergoing lung surgery, (b) controlled primary lesion, (c) no metastasis in organs other than the lungs, or if present, such metastasis could be controlled by surgery or other treatments, and (d) pulmonary metastasis that can be completely removed [17].

## Conclusions

This case emphasises comprehensive evaluations, multidisciplinary discussions, and individualised treatment plans. It encourages patients to remain optimistic and engaged in their healthcare journey. Ensuring the engagement of patients and their caregivers in multidisciplinary tumour board deliberations is of paramount importance. The potential role of surgical intervention in carefully selected cancer survivors of NPC with isolated lung metastases needs to be explored. We would certainly advise caution in deriving generalised conclusions about cures in metastatic NPC from this single exceptional case, and further research is needed to establish guidelines and improve treatment outcomes for this challenging clinical scenario.
